# Intracellular and Intercellular Mitochondrial Dynamics in Parkinson’s Disease

**DOI:** 10.3389/fnins.2019.00930

**Published:** 2019-09-18

**Authors:** Dario Valdinocci, Rui F. Simões, Jaromira Kovarova, Teresa Cunha-Oliveira, Jiri Neuzil, Dean L. Pountney

**Affiliations:** ^1^School of Medical Science, Griffith University, Southport, QLD, Australia; ^2^CNC - Center for Neuroscience and Cell Biology, University of Coimbra, Cantanhede, Portugal; ^3^Institute of Biotechnology, Czech Academy of Sciences, Prague-West, Czechia

**Keywords:** alpha-synuclein, tunneling nanotube, Parkinson’s, mitophagy, mitochondria

## Abstract

The appearance of alpha-synuclein-positive inclusion bodies (Lewy bodies) and the loss of catecholaminergic neurons are the primary pathological hallmarks of Parkinson’s disease (PD). However, the dysfunction of mitochondria has long been recognized as a key component in the progression of the disease. Dysfunctional mitochondria can in turn lead to dysregulation of calcium homeostasis and, especially in dopaminergic neurons, raised mean intracellular calcium concentration. As calcium binding to alpha-synuclein is one of the important triggers of alpha-synuclein aggregation, mitochondrial dysfunction will promote inclusion body formation and disease progression. Increased reactive oxygen species (ROS) resulting from inefficiencies in the electron transport chain also contribute to the formation of alpha-synuclein aggregates and neuronal loss. Recent studies have also highlighted defects in mitochondrial clearance that lead to the accumulation of depolarized mitochondria. Transaxonal and intracytoplasmic translocation of mitochondria along the microtubule cytoskeleton may also be affected in diseased neurons. Furthermore, nanotube-mediated intercellular transfer of mitochondria has recently been reported between different cell types and may have relevance to the spread of PD pathology between adjacent brain regions. In the current review, the contributions of both intracellular and intercellular mitochondrial dynamics to the etiology of PD will be discussed.

## Introduction: PD, α-Synuclein and Mitochondria

The principal histopathological marker of Parkinson’s disease (PD) is the presence in neurons of α-synuclein (α-syn) protein aggregates that occur in inclusion bodies known as Lewy bodies (Shults, 2006; McCann et al., 2014). α-Syn is primarily expressed pre-synaptically and evidence exists of α-syn transfer from neurons to neuronal and non-neuronal cells *in vitro*, indicating that α-syn pathology spreads between anatomically adjacent brain regions by a cell-to-cell transfer mechanism (Valdinocci et al., 2017). α-Syn is a small (14 kDa), acidic protein expressed in the brain, peripheral nervous system and circulating erythrocytes (Thakur et al., 2019). Its pre-synaptic localization and high abundance implicate an important role in synaptic transmission (Burre et al., 2010) with specific functions implicated in synaptic vesicle recycling and regulating soluble NSF attachment protein receptor (SNARE) interactions and dopamine biosynthesis (Theillet et al., 2016; Sulzer and Edwards, 2019). *In vitro* α-syn is a dynamically unfolded protein, although *in vivo* the membrane-associated tetrameric form is proposed to be α-helical (Bartels et al., 2011). Various factors, such as raised copper or calcium concentration, oxidative stress and post-translational modifications can trigger intracellular α-syn aggregation (Fujiwara et al., 2002; El-Agnaf et al., 2006; Reynolds et al., 2008; Rcom-H’cheo-Gauthier et al., 2016). Whilst a definitive link between mitochondrial dysfunction and initiation of PD still does not exist, it is clear that dysfunctional mitochondria are omnipresent in PD (Chen et al., 2019). Moreover, α-Syn can be located at mitochondrial membranes, especially under stress conditions (Cole et al., 2008; Devi et al., 2008), and its aggregation can be linked to mitochondrial dysfunction in PD (Perfeito et al., 2013; Celardo et al., 2014). α-Syn aggregates may in turn cause deleterious alterations in mitochondrial function, including intracellular dynamics. This review focusses on α-syn interactions with mitochondria in PD.

## Intracellular Mitochondrial Dynamics in PD

**FIGURE 1:**
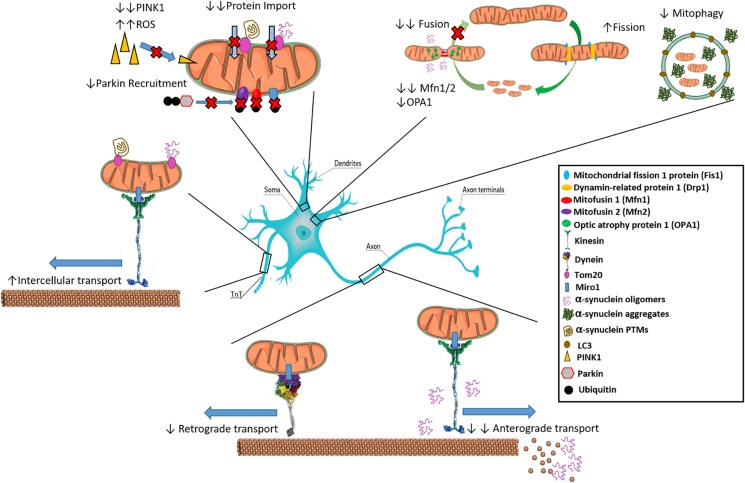
Interactions of α-synuclein with inter- and intra-cellular mitochondrial dynamics. α-syn inhibits mitochondrial fusion as over expression leads to reduced Mfn1/2 and OPA-1. Oligomeric α-syn can interact with kinesin to dislodge it from microtubules preventing anterograde transport along axons. Oligomeric α-Syn also promotes the depolymerization of microtubules, preventing transport of mitochondria. Increased aggregated α-syn is associated with reduced mitophagy. Certain α-Syn types such as oligomers and mutated variants like S129E bind to Tom20 resulting in inhibition of mitochondrial protein import. Reduction in PINK1 leads to inability to signal for mitophagy of α-syn bound mitochondria through Parkin recruitment, leading to ROS generation. Movement of pathological aggregates between cells via TnTs may occur if bound to Tom20 on actively transferring mitochondria.

Mitochondria are highly dynamic and interconnected entities, with the ability to change their morphology in addition to mobilizing within the cell to help power critical functions (Burte et al., 2015). Mitochondrial dynamic processes include fusion/fission, transport and clearance, and are so interconnected and interdependent that they have been proposed to form an interactome that ultimately controls mitochondrial quality, quantity and metabolism (Dorn and Kitsis, 2015; Shirihai et al., 2015). Under normal conditions, mitochondria constantly undergo cycles of fusion and fission that affect their morphology and shape, processes that may be perturbed in PD. α-Syn has been shown to influence mitochondrial size both independently and dependent on fusion/fission proteins, with recent reports detailing these interactions as an attribute of the pathological variants such as oligomers and fibrils, ruling out any negative effects on mitochondrial dynamics as a normal function of the monomer (Wang et al., 2019). Factors such as the GTPases Mitofusin1 and 2 (Mfn1/2), and optic atrophy protein 1 (OPA1) are involved in fusing the outer mitochondrial membrane (OMM) and inner mitochondrial membrane (IMM), respectively, forming elongated structures that are more efficient in ATP generation (Chan, 2012; Tilokani et al., 2018). As illustrated in Figure 1, oligomeric α-syn can bind to lipids in the OMM and distress the membrane curvature, leading to a decrease in mitochondrial fusion rate (Pozo Devoto and Falzone, 2017). In addition, overexpression of α-Syn in transgenic mice led to reductions in Mfn1/2 protein levels, correlating with a decrease in mitochondrial fusion and smaller mitochondria (Xie and Chung, 2012). α-Syn knockdown was shown to trigger mitochondrial elongation (Kamp et al., 2010).

Conversely, mitochondrial fission is dependent on dynamin-related protein 1 (Drp1), mitochondrial fission factor (Mff), mitochondrial fission protein 1 (Fis1) and mitochondrial dynamics proteins of 49 and 51 kDa (MiD49/51) for shrinkage of structures typically promoted during cellular replication (Korobova et al., 2013; Pagliuso et al., 2018). Depending on the isoform, post-translational modification of Drp1 by the small ubiquitin-like modifier (SUMO) drastically alters the effect on mitochondrial fission. SUMO-2/3 for instance has been shown to inhibit Drp1-Mff interaction preventing mitochondrial fragmentation, however, SUMO-1 stabilizes Drp1 leading to enhanced fragmentation; with evidence establishing a link between SUMO and PD, this further implicates mitochondrial dynamics in the pathogenesis of the disease (Guo et al., 2013, 2017; Fu et al., 2014; Vijayakumaran et al., 2015; Henley et al., 2018; Vijayakumaran and Pountney, 2018). Mitochondrial complex I inhibitors, Rotenone and MPP^+^, inducers of parkinsonian phenotypes, were shown to promote mitochondrial fission (Barsoum et al., 2006; Thomas et al., 2011) and Drp1 inactivation prevented the fission phenotype (Wang et al., 2011a). In the substantia nigra of sporadic PD patients, the short form of OPA1 (OPA1-S) was decreased in the absence of changes in Mfn1, further suggesting mitochondrial fusion deficiency (Zilocchi et al., 2018). Thus, a net increase in mitochondrial fission over fusion may ultimately lead to a fragmented mitochondrial network negatively impacting the efficiency of neuronal signaling in PD. Worth noting are reductions in Drp1 in the later stages of degeneration in transgenic mice (Xie and Chung, 2012). Mitochondria are also a major source of intracellular calcium which is released upon electron transport chain dysfunction, such as that caused by rotenone-mediated inhibition, and interacts with α-syn to promote aggregation. Indeed, induction of the endogenous neuronal calcium buffering protein, calbindin-D28k (CB), was able to block rotenone-induced α-syn aggregation and neurons expressing high levels of CB excluded α-syn inclusion bodies in both human and mouse model tissues (Rcom-H’cheo-Gauthier et al., 2016, 2017; McLeary et al., 2019). Moreover, the Miro1 protein acts as a calcium sensor at the mitochondrial outer membrane, reacting to high calcium by augmenting mitophagy (Nemani et al., 2018).

Intracellular mitochondrial dynamics involves the transport of mitochondrial units from one area of the cell to another, and is of key importance in neuronal cells that are polarized with long axons and dendrites (Lin and Sheng, 2015). Mitochondria need to be transported to synaptic terminals, active growth cones and axonal branches, where they maintain energy and Ca^2+^ homeostasis (Sheng, 2017). Trafficking of mitochondria is microtubule (MT)-based, relying on ATP and motor proteins. MTs are polar α/β-tubulin polymers with the minus end within the cell body and the plus end to the cell extremity (Tas and Kapitein, 2018), and neurons can move mitochondria in both directions using independent motor proteins. Kinesins drive transport toward the cell extremities (anterograde transport) whereas dynein motors are responsible for retrograde transport (Hirokawa et al., 2010). Hydrolysis of ATP is essential to fuel mitochondrial movement in both directions (Zala et al., 2013). Bridging between mitochondria and MT-bound motor proteins are motor adaptor proteins such as Trafficking Kinesin Proteins 1 and 2 (TRAK1 and 2), which connect the OMM protein Mitochondrial Rho GTPase 1 (Miro1) to kinesin (Melkov and Abdu, 2018). Indeed, Miro1 also mediates Drp1/Fis1-independent mitochondrial shape transition (Mist), needed for mitophagy (Nemani et al., 2018). Syntabulin can also link mitochondria and kinesins whilst syntaphilin acts as an anchor stopping mitochondrial movement (Cai et al., 2005; Chen and Sheng, 2013).

An increase in α-syn concentration was shown to result in mitochondrial traffic arrest even before axonal degeneration, affecting both anterograde and retrograde transport (O’Donnell et al., 2014; Pozo Devoto and Falzone, 2017). Nigral dopaminergic neurons especially display a “dying back” pattern, wherein anterograde trafficking of mitochondria becomes disrupted in early stage sporadic PD followed by retrograde transport at late stage (Chu et al., 2012). Loss of this trafficking severely reduces nigral neuron ability to regulate the conditions necessary for axonal signaling and neurotransmitter release at terminals. Regarding anterograde trafficking, α-syn oligomers disrupt through direct binding interactions between kinesin and the MT in addition to increasing expression of tau, a MT structure disruptor (Prots et al., 2013, 2018). α-Syn appears to induce MT fragmentation directly as well, hindering mitochondrial movement from distal cell areas (Melo et al., 2017). Interestingly, this finding produced the opposite outcome to Chu et al. (2012) whereby retrograde transport was disrupted first. This may be due to differences in α-syn species interaction with trafficking complexes disrupting mitochondrial transport as Chu et al. (2012) brought about “dying back” through viral overexpression of α-Syn whilst Melo et al. (2017) utilized an A53T transgenic model. Moreover, the PD-linked protein leucine rich repeat kinase-2 (LRRK2) seems to alter MT polymerization/depolymerization cycles, affecting mitochondrial trafficking (Gillardon, 2009; Godena et al., 2014). Impairment of mitochondrial transport is also induced by the parkinsonian toxin MPP^+^, which inhibits kinesin-1-mediated anterograde transport leading to an increase in dynein-dependent retrograde transport (Morfini et al., 2007). LRRK2 and PTEN-induced kinase 1 (PINK1) mutant drosophila also exhibit disturbed mitochondrial calcium homeostasis with functional involvement of Miro1 (Lee et al., 2018).

## Role of Mitophagy in PD

Mitochondrial dynamics also includes mitochondrial clearance by mitophagy, a mitochondrion-specific autophagic process that drives dysfunctional mitochondria to degradation in autophagosomes (Rodolfo et al., 2018). Several mitophagy mechanisms have been described, which can be dependent on or independent of mitochondrial receptors (Martinez-Vicente, 2017), including B-cell lymphoma 2 nineteen kilodalton interacting protein 3 (BNIP3), Nix, Bcl-2-like protein 13 (Bcl2-L-13) and Fun14 domain-containing protein 1 (FUNDC1), which interact with microtubule-associated proteins 1A/1B light chain 3B (LC3), recruiting the autophagosomal machinery (Chu, 2018). Cardiolipin may also mediate mitophagy, driving mitochondrial degradation when this phospholipid (that has a LC3-binding motif) moves from the IMM to the OMM (Chu et al., 2013). Receptor independent mitophagy involves the priming of mitochondria after PINK1 translocation from the cytosol to the mitochondria. The loss of mitochondrial membrane potential, and inhibition of PINK1-degrading proteases, leads to PINK1 accumulation in mitochondria, where it recruits the E3 ubiquitin ligase Parkin that initiates mitophagy by ubiquitinating OMM proteins, such as Mfn1 and Mfn2, Miro1, translocase of outer mitochondrial membrane 20 (TOM20), and voltage-dependent anion channel (VDAC) (Meissner et al., 2015). Poly-ubiquitinated OMM proteins act as mediators that ultimately cause mitochondrial engulfment by autophagosomes (Yoshii and Mizushima, 2015).

Evidence for a role of mitophagy in PD includes the observation that α-syn overexpression decreases the level of LC3-positive vesicles in human neuroblastoma cells (Winslow et al., 2010). Interestingly, mutations in PINK1 and Parkin genes cause autosomal recessive forms of familial early onset PD (Valente et al., 2001; Mata et al., 2004; Kumar et al., 2017), implicating a role of mitophagy in the aetiopathogenesis of PD (Dawson and Dawson, 2010). Mitochondrial morphology aberrations were found in a PINK1-mutant Drosophila model, and overexpression of Parkin was shown to rescue the phenotype (Clark et al., 2006). The role of Parkin mutations in mitophagy impairment was confirmed using iPSC-derived dopaminergic neurons with mutations in the Parkin gene (Suzuki et al., 2017). Additionally, Parkin has been demonstrated to be highly insoluble in PD, compromising autophagic systems (Lonskaya et al., 2013). Increased pathological α-syn leads to increased cytosolic Ca^2+^ and Miro1 upregulation, however, the adaptor function of Miro1 between mitochondria and motor transport complexes is abrogated at high Ca^2+^ concentration (Saotome et al., 2008; MacAskill et al., 2009; Wang and Schwarz, 2009). In addition to transport, increased Miro1 protects mitochondria from mitophagy. Thus, in PD it is likely that Miro1 upregulation in combination with PINK1 reduction serves to delay degradation allowing for unregulated ROS generation (Shaltouki et al., 2018). Interestingly, an increase in Miro1-dependent anterograde transport of mitochondria was found in a PINK1-knockout model (Liu et al., 2012). It is perhaps possible that in the early stages of PD, whilst cytosolic Ca^2+^ is low, that the transport function of Miro1 is unaffected, then changing to act as a protector especially in later stages. Phosphorylation of Miro1 by PINK1 is required for Miro1 degradation, however, mutations in PD likely affect this function (Wang et al., 2011b; Shlevkov et al., 2016). In combination with the protective capabilities of Miro1, this may explain why there is a frequent accumulation of dysfunctional mitochondria in distal axonal areas in PD models whilst still ubiquitinated by Parkin. Recent work by Grassi et al. (2018) suggests the possibility of differing α-syn variants affecting different cellular systems, with a non-fibrillar phosphorylated α-syn species described to induce mitophagy. However, understanding of mitophagy in PD is relatively limited due to the lack of amenable *in vivo* experimental approaches.

## Intercellular Transfer of Mitochondria and α-Synuclein

Genes move from progenitors to progeny, i.e., in a vertical manner, while horizontal gene transfer (HGT) is rare among eukaryotes (Keeling and Palmer, 2008; Davis et al., 2014; Davis and Xi, 2015). Recently, HGT has been reported for mitochondrial genes, via horizontal transfer of mitochondria between cells *in vitro* (Rustom et al., 2004; Spees et al., 2006; Rogers and Bhatacharya, 2013; Ahmad et al., 2014; Wang and Gerdes, 2015; Rustom, 2016; Sinha et al., 2016). Wang and Gerdes (2015) showed that organelles, including mitochondria, move between cells via so-called tunnelling nanotubes (TNTs), narrow inter-cellular bridges with actin as a structural protein and with tubulin fibers as “tracks” for movement of subcellular structures between cells (Rustom et al., 2004). Co-culture studies showed that transfer of mitochondria from mesenchymal stem cells (MSCs) into cancer cells with defects in mitochondrial DNA (mtDNA) resulted in recovery of mitochondrial respiration in cancer cells (Spees et al., 2006) and mitochondria, moving from healthy cells via TNTs, rescued cancer cells exposed to mitochondrial insults during early stages of apoptosis (Wang and Gerdes, 2015). Mice with experimental lung disease were grafted with allogenic MSCs with labeled mitochondria resulting in movement of the mitochondria to the diseased cells, recovering their respiration and alleviating the pathology (Islam et al., 2012). Inter-cellular transfer of mitochondria maintains balanced heteroplasmy of mtDNA in outbred individuals (Jayaprakash et al., 2015), pointing to mitochondrial HGT as a more frequent event than previously considered (Berridge et al., 2015, 2016).

In relation to disease, transfer of damaged mtDNA has been observed in various mouse models of engrafted tumor cells with other works such as that by Dong et al. (2018) revealing that damaged mtDNA is transported within the mitochondria (Tan et al., 2015). Such a process allows for the damaged mtDNA to affect the acceptor cell through the generation and leakage of ROS, especially so if mitophagy or systems regulating the prevention of damaged mitochondrial spread are faulty. Regarding PD, knowledge on mtDNA and mitochondrial spread in general is still unclear. mtDNA mutations are observed in neuronal cells in early stages of PD (Braak Stage 3 onward) due to damage attained via oxidative stress (Lin et al., 2012). This is not observed in late stage PD. However, this could potentially be due to the increased chances of neurons that previously hosted the damaged mtDNA of being destroyed, in addition to a lower population of neurons with damaged mtDNA prior to death. Various interactions of α-syn with mitochondria lead to the generation of ROS and thus increase risk of damaged mtDNA, including certain pathological α-syn species shown to bind with high affinity to the Tom20 mitochondrial outer membrane protein, thereby inhibiting mitochondrial protein uptake and promoting ROS generation (Di Maio et al., 2016; Grassi et al., 2018). Recent work by Wang et al. (2019) further reveals α-syn bound mitochondria only occurs with pathological α-syn aggregate species and not physiological monomers. This finding did not pertain only to PD but other synucleinopathies such as Dementia with Lewy Bodies and Multiple System Atrophy. In addition α-syn is also shown to inhibit Complex I directly, further compromising the energy production of the mitochondrion and increasing the generation of ROS (Reeve et al., 2015). No evidence has emerged to suggest that α-syn-affected mtDNA or mitochondria can spread to healthy cells, however, further investigation is required.

Mitochondrial transport is driven by motor complexes via binding through specific adaptor proteins, such as Miro1, suggesting that a potential mechanism of mitochondrial transfer between cells is mediated via the motor systems using TNTs (Ahmad et al., 2014; Sinha et al., 2016). This is based on experimental data (Ahmad et al., 2014) as well as analogy with the movement of mitochondria along axons in neuronal cells, where the kinesin and dynein motor systems include the motor protein plus two adaptor proteins Milton and Miro1, the latter with high affinity for mitochondria (Hase et al., 2009; Wang and Schwarz, 2009; MacAskill and Kittler, 2010; Kimura et al., 2012; Schiller et al., 2013). Indeed, mutations in the Miro1 gene, RHOT1, have recently been linked to PD cases, wherein the mutations led to fewer ER-mitochondria contact sites, calcium dyshomeostasis and exacerbation of calcium-dependent mitochondrial fragmentation and increased mitochondrial clearance (Grossmann et al., 2019). How the movement of mitochondria between cells is triggered and regulated is still not fully understood. Recent work has shown α-syn utilizing TNTs for spread. Thus, Dieriks et al. (2017) demonstrated the establishment of TNTs and transport of α-syn from neurons and pericytes in culture. Interestingly, Rostami et al. (2017) revealed TNT formation and α-syn transfer between astrocytic cells, transport of healthy mitochondria to rescue stressed mitochondria damaged by α-syn and transport of α-syn from α-syn affected astrocytes to healthy astrocytes. There were no reports of α-syn utilizing mitochondria for TNT-mediated migration, however, mitochondria may also represent an efficient carrier of α-syn between certain cell types. Furthermore, prion proteins, which have some similarities in mode of propagation to α-syn aggregates, have been established to hijack TNTs to mediate cell-to-cell transfer of the infectious protein (Gousset et al., 2009; Dorban et al., 2010; Prusiner et al., 2015; Steiner et al., 2018). Although there are many potential mechanisms of α-syn spread (Valdinocci et al., 2017), in view of findings that α-syn can migrate within TNTs bound to organelles such as lysosomal vesicles (Abounit et al., 2016) it is tempting to speculate that α-syn may transfer between neighboring cells bound to mitochondria that are translocated actively along TNTs (Figure 1).

## Potential for Mitochondrially Targeted PD Therapeutics

The influences of pathological α-syn on mitochondrial dynamics in PD are potentially wide-ranging. Mitochondria-targeted drugs, such as Mito-Q and Mito-Apocynin showed therapeutic potential in experimental PD models whilst others such as Metformin are variable likely attributed to model utilized (Patil et al., 2014; Bayliss et al., 2016; Ismaiel et al., 2016; Lu et al., 2016; Langley et al., 2017; Xi et al., 2018). Although therapies that target calcium mobilization or oxidative stress tackle the effects of mitochondrial dysfunction, future innovative approaches could ameliorate mis-location of mitochondria or intercellular mitochondrial transfer. Further work is clearly needed to articulate the full significance of changed mitochondrial dynamics in PD etiology.

## Author Contributions

All authors have contributed to the preparation of the manuscript, with DP taking responsibility for editing the final version of the manuscript.

## Conflict of Interest Statement

The authors declare that the research was conducted in the absence of any commercial or financial relationships that could be construed as a potential conflict of interest.
